# Performance of administrative databases for identifying individuals with multiple sclerosis

**DOI:** 10.1038/s41598-023-45384-w

**Published:** 2023-10-25

**Authors:** Pauline Ducatel, Marc Debouverie, Marc Soudant, Francis Guillemin, Guillaume Mathey, Jonathan Epstein

**Affiliations:** 1 Department of Neurology, Nancy University Hospital, 29 Avenue du Maréchal de Lattre de Tassigny, Nancy, France; 2 CIC-EC 1433, CHRU, Inserm, Université de Lorraine, 9 Av. de La Forêt de Haye, Vandoeuvre-Lès-Nancy, France; 3 Université de Lorraine, EA 4360 Apemac, 9 Av. de La Forêt de Haye, Vandoeuvre-Lès-Nancy, France

**Keywords:** Multiple sclerosis, Epidemiology

## Abstract

Administrative databases are an alternative to disease registries as a research tool to study multiple sclerosis. However, they are not initially designed to fulfill research purposes. Therefore, an evaluation of their performance is necessary. Our objective was to assess the performance of the French administrative database comprising hospital discharge records and national health insurance databases in identifying individuals with multiple sclerosis, in comparison with a registry that exhaustively compiles resident multiple sclerosis cases in Lorraine, northeastern France, as reference. We recorded all individuals residing in the Lorraine region who were identified by the administrative database or the registry as having multiple sclerosis from 2011 to 2016. We calculated the Matthews correlation coefficient and other concordance indicators. For identifying individuals with multiple sclerosis, the Matthews correlation coefficient by the administrative database was 0.79 (95% CI 0.78–0.80), reflecting moderate performance. The mean time to identification was 5.5 years earlier with the registry than the administrative database. Administrative databases, although useful to study multiple sclerosis, should be used with caution because results of studies based on them may be biased. Our study highlights the value of regional registries that allow for a more exhaustive and rapid identification of cases.

## Introduction

Multiple sclerosis (MS) is a neuroinflammatory disorder affecting the central nervous system. This disease has high socio-economic impact^1,2^ and is considered a public health priority in many countries.

Disease registries provide researchers and policymakers with accurate information to conduct observational study, monitor conditions and plan healthcare^3,4^. However, they require substantial resources and careful planning^5^. Therefore, administrative databases (ADs) represent an alternative tool that may be preferred.

ADs are designed for financial and administrative purposes and are increasingly used in medico-economic studies or pharmacoepidemiology or epidemiological research^6^. Recent MS descriptive studies have used ADs for both prevalence and incidence estimates^7–9^. In France, the AD combines hospital discharge records and national health insurance databases. It recognizes MS cases by the presence of at least one of 1) reimbursement for a disease-modifying treatment considered specific to this condition, 2) declared disease-specific payment exemption for MS (in France, this registration allows patients with a particular illness to be 100% reimbursed for their outpatient and inpatient health care costs) and/or 3) hospital discharge with MS diagnosis.

However, the registered prevalence of disease-specific payment exemptions has been found systemically lower than the actual prevalence of illnesses^10^. MS exemptions can also be misreported for a related condition^11^. Moreover, benign MS cases do not necessarily require the initiation of disease-modifying therapy or hospitalization. Indeed, the number of cases without treatment is estimated at 31%^12^. Furthermore, the accuracy of coding diagnoses in the hospital discharge records for MS is uncertain^13,14^. Therefore, the use of ADs outside the designed financial and administrative objectives^6,15^ could lead to a misclassification of individuals with MS and affect the conclusions of studies relying on these databases.

To determine the reliability of a data source, one of the most commonly used methods is comparison with a reference of recognized validity^16^. The *Registre Lorrain de la Sclérose en plaques* (ReLSEP) collects MS cases in the Lorraine region, in northeastern France. It was created to meet medical research and MS incidence monitoring objectives. To identify cases, ReLSEP relies on the reports of all neurologists in the region who are an essential part of patients’ healthcare pathways. Moreover, multiple sources are also solicited, including the AD.

The main objective of this study was to assess the performance of the French AD in identifying individuals with MS in comparison with the ReLSEP reference. Our secondary objectives were to determine factors associated with misidentification by the AD; assess and compare the proportion of cases recognized for the first time by ReLSEP or the AD, among cases identified in common; and ascertain the performance of different combinations of criteria to identify MS cases in ADs.

## Materials and methods

### Design

We conducted a study targeting residents in the Lorraine region (comprising four departments: Meurthe-et-Moselle, Meuse, Moselle and Vosges). Cases were identified by the AD or ReLSEP as MS from January 1, 2011 to December 31, 2016. This time frame was chosen to allow for homogeneity in AD and ReLSEP identification criteria, as the MS 2010 McDonald criteria^17^ were modified in 2017^18^. Also, we determined in previous analyses that a newly diagnosed case of multiple sclerosis was identified by the ReLSEP within 5 years^19^. Therefore, the end of the study period was set 5 years previous to this study analysis to provide sufficient hindsight to enable a reliable identification of MS cases and comparison of the identification time between the ReLSEP and AD.

### ReLSEP

The ReLSEP was created in 2003. It exhaustively compiles and verifies all MS cases of patients residing in Lorraine. It relies on the reports of all neurologists in the region (hospital and private practice). It also benefits from an annual extraction from multiple sources: the AD, the two regional biology laboratories that analyze cerebral spinal fluid (CSF) samples and two regional networks of MS professionals (LORSEP and ALSASEP).

To ensure the accuracy of the diagnosis, each case is confirmed by a neurologist specialized in MS and is validated only if the 2010 McDonald criteria are met^17^.

### AD

Data are collected annually from the hospital discharge records and the national health insurance databases and are nominative. National health insurance data contain two criteria: reimbursement for a disease-modifying treatment considered specific to MS and MS exemption. For each health institution in France (including outside Lorraine), hospital discharge data for patients residing in Lorraine related to a MS diagnosis are transmitted annually.

Data from annual extractions are cross-referenced with data for individuals already in the ReLSEP and matched by last name, first name, date of birth, sex and department of residence. The search for duplicates is performed using a semi-manual method. When files match on all criteria, they are considered as duplicates. In case of doubt, a source check can be carried out to conclude.

Each reported case is subjected to a validation procedure before being included in the ReLSEP. The patient’s file is retrieved from the general practitioner or specialist by clinical research nurses. A neurologist specialized in MS validate the diagnosis if the 2010 McDonald criteria are met.

### Data collected

For the individuals in the registry, the variables of interest were age at onset, sex, department of residence, first healthcare facility, associated comorbidities, first Expanded Disability Status Scale (EDSS) assessing the level of disability, number of MS relapses over the first 2 years after diagnosis, results of the first brain MRI, cerebral spinal fluid analysis performed and initial treatment. Data regarding individuals known only to the AD were limited to available data: age at identification by the AD, sex, department of residence, year of identification by the AD and first healthcare facility (Supplementary information SI1).

### Statistical analysis

The individuals recognized by the two sources were true positives (TPs). We defined as false positives (FPs) the individuals detected by the AD but not the ReLSEP, and as false negatives (FNs), the individuals identified by the ReLSEP but not the AD. The true negatives (TNs) referred to the Lorraine’s population and were estimated from data reported annually by the French national institute for statistical and economic studies^20^.

We chose the Matthews correlation coefficient (MCC) as the primary endpoint^21^. The MCC corresponds to the Pearson correlation coefficient applied to a binary classification and is interpreted in the same way^22,23^. It is popular in the machine learning field^24^ and has previously been used to evaluate the performance of ADs^25^. It is a useful metric for unbalanced datasets and allows for considering all the parameters of the contingency table^26^. The MCC ranges from − 1 to + 1, indicating perfect agreement (+ 1) or disagreement (-1), and 0 means no relationship. A correlation ≥ 0.8 is considered strong and below moderate^27^.

Categorical variables were described with number and percentage and quantitative variables with mean and standard deviation.

The primary analysis included the calculation of the MCC and other indicators: sensitivity (Se), specificity (Sp), positive predictive value (PPV), negative predictive value (NPV), accuracy, Cohen’s kappa and F1-score. We used bootstrapping to compute the confidence intervals of the F1-score and MCC.

Using the TPs as a reference, we compared the TPs and FPs, and the TPs and FNs. For the bivariate analyses, Kruskal–Wallis, chi-square, Wilcoxon and Fisher’s exact test were used as appropriate, with a 0.05 two-sided significance level.

For the multivariable analyses, we used three hierarchical logistic regression models with the department of residence as a random variable and, when appropriate, the year of identification by the AD (Supplementary Information SI2). The first model studied factors associated with misidentification as MS cases by the AD. The second model investigated factors associated with failure to identify MS cases by the AD. For these two multivariate models, we retained factors with *p* < 0.15 on bivariate analysis. Age and sex were also included because of their role in the use of healthcare described in the literature^28,29^. Odds ratios and 95% confidence intervals (CIs) were estimated. We calculated the time to identify individuals with MS by the ReLSEP and AD. With a third model, we examined factors associated with early identification by the AD versus the ReLSEP among TPs. Because the AD is extracted annually, cases were considered identified first by the ReLSEP if they were recognized at least 1 year before the AD. This was the most unfavorable situation for the registry.

We used the VIM package to provide a visual representation of the proportion of missing data for each variable, as well as their distribution (Supplementary information SI3, SI4 and SI5). Multiple imputations were performed under the missing at random assumption, using the predictive mean matching method of the MICE package^30^ (15 imputations). The variables imputed were the outcome and covariates with missing data. After generating imputed datasets, we ran our models using the with() function on each dataset. Finally, we pooled the results over the imputed datasets using the pool() function to compute multilevel (residence + /− year of identification by the AD as random variables) imputed (average effect relative to each imputed dataset) odds ratio. We also conducted complete-case sensitivity analyses to confirm the results of our secondary analyses.

To assess the performance of different combinations of the three criteria (reimbursement for a disease-modifying treatment, MS exemption and hospital discharge) for identifying MS cases using the AD, we calculated, for each combination, the same metrics used beforehand.

All analyses were performed with R 4.1.2.

### Ethical approval and Informed consent

The National Commission for Data Protection and Liberties and the Consultative Committee for the data processing in health research gave both a favorable opinion for the collection of data by the RELSeP (no. 913001 the 01/06/2014 and no. 10–258 the 05/06/2010). All methods were performed in accordance with the relevant guidelines and regulations. Informed consent was obtained from all subjects and/or their legal guardian(s).

## Results

Of the 2084 individuals identified by ReLSEP, 1152 (55%) were declared by neurologists/LORSEP, 759 by biology laboratories (36.2%) and 37 by ALSACEP (1.8%).

During the considered period, 1826 cases were TP, 258 were FN and 725 were FP. In total, 28.4% (725/2551) of individuals reported by the AD were wrongly identified as having MS. The estimated number of TNs was 2,245,196 (Supplementary information SI6).

The characteristics of TPs and FNs are in Table 1 (and Supplementary information SI7). The variable with the highest rate of missing data was the first EDSS (8.5%). For FPs, the mean age at identification by the AD was 50.8 (18.6) years, 68.5% were women. Most patients resided in Moselle (49.8%) and were followed in a hospital (80.2%) (Supplementary information SI8).

**Table 1:** Factors associated with failure to identify multiple sclerosis by the administrative database.

Status		TP	FN	OR (multilevel imputed) (95% CI, *p* value)
		N = 1826	N = 258	
Age at onset, years	Mean (SD)	33.4 (11.4)	33.5 (12.0)	1.01 (0.99–1.02, *p* = 0.460)
Sex	Male	538 (29.8)	66 (25.6)	–
	Female	1270 (70.2)	192 (74.4)	1.05 (0.77–1.45, *p* = 0.749)
First healthcare facility	Hospital	1409 (77.4)	215 (83.3)	–
	Private practice	411 (22.6)	43 (16.7)	0.72 (0.50–1.04, *p* = 0.078)
Associated comorbidities	No	611 (33.5)	104 (40.3)	–
	Yes	1215 (66.5)	154 (59.7)	0.85 (0.63–1.13, *p* = 0.266)
First EDSS	Mean (SD)	2.7 (2.0)	1.8 (1.6)	0.68 (0.61–0.75, *p* < 0.001)
Number of relapses over the first 2 years	Mean (SD)	1.2 (0.9)	1.1 (0.6)	0.73 (0.59–0.91, *p* = 0.005)
First brain MRI	Normal	83 (4.7)	28 (10.9)	–
	Abnormal	1678 (95.3)	229 (89.1)	0.47 (0.28–0.76, *p* = 0.002)
CSF analysis performed	Yes	1312 (71.9)	208 (80.6)	–
	No	514 (28.1)	50 (19.4)	0.73 (0.51–1.05, *p* = 0.089)
Initial treatment	None	315 (17.3)	88 (34.1)	–
	Moderate	1211 (66.3)	139 (53.9)	0.33 (0.23–0.46, *p* < 0.001)
	Highly	300 (16.4)	31 (12.0)	0.49 (0.30–0.79, *p* = 0.003)

Number (multilevel imputed): 2084, EDSS = Expended Disability Status Scale, CSF = cerebrospinal fluid, TP = false positive, FN = true negative, OR = odds ratio, 95% CI = 95% confidence interval.

The concordance indicators assessing the performance of the AD are summarized in the last row of Table 2. The MCC was 0.79 (95% CI 0.78–0.80), reflecting moderate performance by the AD in identifying MS cases. Other global metrics such as kappa (0.79 [95% CI 0.78–0.80]) and F1-score (0.79 [95% CI 0.77–0.80]) confirmed this finding. The Sp, NPV and accuracy were 0.99 (95% CI 0.99–0.99). The Se was 0.88 (95% CI 0.86–0.99) and PPV 0.72 (95% CI 0.70–0.73). Because most of the FPs and FNs resided in Moselle, we performed a sensitivity analysis by removing individuals from Moselle (Supplementary Information SI9). The results of the main analysis were not modified, suggesting that because Moselle is Lorraine's most populous department (almost half of Lorraine's population), the largest number of VPs, FPs and FNs reside there.

**Table 2:** Performance of different combinations of administrative database criteria to identify individuals with multiple sclerosis.

Criteria	Sensitivity (95% CI)	PPV (95% CI)	F1-score (95% CI)	Kappa (95% CI)	MCC (95% CI)
DMT	0.38 [0.36–0.40]	0.86 [0.84–0.88]	0.53 [0.51–0.55]	0.53 [0.51–0.55]	0.57 [0.55–0.59]
DSPE	0.58 [0.56–0.60]	0.82 [0.80–0.84]	0.68 [0.66–0.70]	0.68 [0.66–0.70]	0.69 [0.67–0.71]
DMT + DSPE	0.61 [0.59–0.63]	0.80 [0.78–0.82]	0.69 [0.67–0.71]	0.69 [0.67–0.71]	0.70 [0.68–0.71]
HD	0.68 [0.66–0.70]	0.74 [0.72–0.76]	0.71 [0.69–0.73]	0.71 [0.70–0.72]	0.71 [0.69–0.73]
DMT + HD	0.79 [0.78–0.81]	0.74 [0.72–0.76]	0.77 [0.75–0.78]	0.77 [0.75–0.78]	0.77 [0.75–0.78]
DSPE + HD	0.87 [0.85–0.88]	0.73 [0.71–0.74]	0.79 [0.78–0.80]	0.79 [0.78–0.80]	0.79 [0.78–0.81]
DMT + DSPE + HD	0.88 [0.86–0.89]	0.72 [0.70–0.73]	0.79 [0.77–0.80]	0.79 [0.78–0.80]	0.79 [0.78–0.80]

DMT = disease-modifying treatment, DSPE = disease-specific payment exemption, HD = hospital discharge, PPV = positive predictive value, MCC = Matthews correlation coefficient, 95% CI = 95% confidence interval.

Table 2 also summarizes the performance of different combinations of the three identification criteria in the AD. When using unique criteria, hospital discharges had the best Se (0.68 [range 0.38–0.68]) and reimbursement for a disease-modifying treatment the best PPV (0.86 [range 0.74–0.86]). The addition of reimbursement for a disease-modifying treatment to the combination of the two other criteria did not bring any benefit for all metrics. Unlike FPs, most TPs were identified by 2 or 3 criteria in the AD. The HD criterion detected the greatest number of TPs and FPs, while the DMT criterion identified the fewest (Supplementary information SI10).

The only factor associated with probability of FP as compared with TP was age (OR = 1.03 [95% CI 1.02–1.04] for 1 more year) (Table 3).

**Table 3:** Factors associated with misidentification as multiple sclerosis by the administrative database.

Status		TP	FP	OR (multilevel imputed) (95% CI, p value)
		N = 1826	N = 725	
Age at onset, years	Mean (SD)	43.4 (15.0)	50.8 (18.6)	1.03 (1.02–1.04, *p* < 0.001)
Sex	Male	538 (29.8)	219 (31.5)	–
	Female	1270 (70.2)	476 (68.5)	0.98 (0.80–1.19, *p* = 0.827)

Number (multilevel imputed): 2551, TP = true positive, FP = false positive, OR = odds ratio, 95% CI = 95% confidence interval.

MS cases with factors reflecting benign forms ^31^ were less likely to be identified by the AD. Indeed, the following characteristics were associated with the probability of FN: low EDSS (OR = 1.49 [95% CI 1.33–1.64] for 1 unit decrease), low number of MS relapses over the first 2 years after diagnosis (OR = 1.35 [95% CI 1.10–1.79] for 1 unit decrease), first brain MRI normal (abnormal OR = 0.46 [95% CI 0.28–0.76]) and no treatment initially (moderate activity OR = 0.33 [95% CI 0.23–0.45] and high activity OR = 0.49 [95% CI 0.30–0.79]) (Table 1).

Among MS cases identified by the two sources, 60.5% were identified first by the ReLSEP. The mean time to identification was 5.5 years earlier with the ReLSEP than the AD (median 1 year) (Supplementary information SI11). Factors associated with early identification by the AD are in Table 4.

**Table:** Factors associated with early identification of individuals with multiple sclerosis by the administrative database.

First identification		ReLSEP	AD	OR (multilevel imputed) (95% CI, *p* value)
		N = 1094	N = 714	
Age at onset, years	Mean (SD)	33.5 (11.5)	33.1 (11.3)	1.00 (0.99–1.01, *p* = 0.781)
Sex	Male	301 (27.5)	237 (33.2)	–
	Female	793 (72.5)	477 (66.8)	0.69 (0.54–0.88, *p* = 0.002)
First healthcare facility	Hospital	799 (73.2)	596 (83.6)	–
	Private practice	293 (26.8)	117 (16.4)	0.58 (0.44–0.76, *p* < 0.001)
Associated comorbidities	No	365 (33.4)	228 (31.9)	–
	Yes	729 (66.6)	486 (68.1)	1.02 (0.81–1.29, *p* = 0.851)
First EDSS	Mean (SD)	2.9 (2.1)	2.4 (1.8)	1.04 (0.96–1.13, *p* = 0.348)
Number of relapses over the first 2 years	Mean (SD)	1.2 (0.9)	1.3 (0.9)	1.09 (0.94–1.26, *p* = 0.260)
Form of MS	RR	647 (59.2)	576 (80.9)	–
	PP	170 (15.6)	108 (15.2)	0.51 (0.32–0.81, *p* = 0.005)
	SP	276 (25.3)	28 (3.9)	0.08 (0.05–0.13, *p* < 0.001)
First brain MRI	Normal	70 (6.6)	13 (1.9)	–
	Abnormal	990 (93.4)	688 (98.1)	4.16 (2.22–7.82, *p* < 0.001)
CSF analysis performed	Yes	721 (65.9)	591 (82.8)	–
	No	373 (34.1)	123 (17.2)	0.50 (0.38–0.66, *p* < 0.001)
Initial treatment	None	211 (19.3)	86 (12.0)	–
	Moderate	716 (65.4)	495 (69.3)	1.59 (1.14–2.21, *p* = 0.007)
	Highly	167 (15.3)	133 (18.6)	3.06 (2.02–4.64, *p* < 0.001)

Number (multilevel imputed): 1826 (the first source of identification was missing for 18 true positives), MS = multiple sclerosis, EDSS = Expended Disability Status Scale, CSF = cerebrospinal fluid, AD = administrative database, ReLSEP = *Registre Lorrain de la Sclérose en plaques*, RR = relapse remitting, PP = primary progressive, SP = secondary progressive, OR = odds ratio, 95% CI = 95% confidence interval.

The findings obtained with complete-case analyses did not differ from multiple imputation, reinforcing and strengthening the results of our secondary analyses (Supplementary information SI12, SI13 and SI14).

## Discussion

The performance of the AD for identifying individuals with MS in comparison with the ReLSEP was moderate, with an MCC of 0.79. This finding may have consequences for the results of the studies using ADs, depending on their objectives. A previous study conducted in 2012 by Foulon et al.^9^ investigated the prevalence of MS in each region of France using the AD. As an illustration, one can correct the prevalence observed in this study according to the sensitivity and specificity obtained in our study^32^. For the highest (lowest) regional prevalence observed at 200.2 (125.6) per 100 000 inhabitants, the corrected prevalence would be 191.8 (105.9). The total prevalence in France would be overestimated by 11.3% (151.2 vs 135.8 per 100 000 inhabitants). Thus, based solely on the AD, the MS prevalence would be overestimated, varying from 4.4% to 18%. Also, individuals with benign MS were more likely to be missed by the AD, because they use less healthcare. This finding could affect the results of medico-economic studies, for example, by overestimating the average costs per patient or misestimating the costs of the disease^33–35^. Similarly, the results of any study in which differential identification of the most severe forms may have an impact could be affected. Moreover, we determined that among the mutual cases known by both sources, 60% were identified by the ReLSEP at least 1 year before the AD. Thus, not only were not all individuals with MS identified by the AD, they were also known later than by ReLSEP. This observation challenges the preconceived notion that ADs allow for a timely monitoring of the disease^36^ and raises the question of their relevance for incidence studies^7,37,38^.

Interestingly, the FPs far outnumbered the FNs. These FPs correspond to individuals identified by the administrative database but whose diagnosis was ultimately refuted after study of their file by a neurologist specialized in MS. Apart from misdiagnosis, several hypotheses can be put forward to explain these FPs. It can happen that a disease-specific payment exemption for MS is wrongly declared when a patient suffers from a condition similar to MS that does not belong to the list of disease-specific payment exemption^14^. In addition, uncertainty about the quality of diagnosis coding for MS in the hospital discharge records has been described previously^11^. Unfortunately, we cannot provide more information regarding the FPs and the reasons behind misidentification. As these individuals do not have MS, they are not part of the ReLSEP and we are unable to collect data about them.

Several countries have conducted validation studies of algorithms used to detect individuals with MS in ADs. The criteria used were close to those of the French AD: mainly inpatient/outpatient encounters^39–41^, more rarely drug dispensing records as well or even disease-specific payment exemption^42,43^. However, the chosen references were imperfect in exhaustively identifying MS cases. Indeed, some verified the MS diagnosis of individuals identified by ADs from medical records^39,44^. Therefore, this strategy does not adequately detect the FNs missed by ADs. Additional studies used regional registries populated by one^45^ or more^42,43^ specialized regional care centers. Nevertheless, in contrast with individuals in the ReLSEP, those not followed up in these institutions could not be identified by the reference. Other works were based on registries in which physician participation was voluntary^40,41^, with a diagnosis not always confirmed by a neurologist^41^.

The ReLSEP represents the main strength of this work. This registry exhaustively identified validated MS cases in Lorraine. It had the advantage of providing detailed information about clinical elements, complementary examinations, treatments and follow-up, impossible to collect from ADs. Also, the study period selected allowed for reliable identification of individuals with MS with a sufficient hindsight of more than 5 years, consistent with the chronic and progressive nature of the disease. Finally, the combination of the three criteria of the AD considered in our study corresponded to their use in practice in identifying MS cases in France^9,33,46^. To strengthen our external validity, other combinations of criteria, which may more closely approximate the use of ADs in other countries, were assessed.

The AD, whose performance was evaluated in our study, is also part of the ReLSEP sources. This situation could have led to incorporation bias, which can occur when the test under consideration is used to determine the reference. This type of bias is likely to overvalue the performance of the studied procedure^47^. Yet, despite this potential overestimation, the performance of the AD was moderate, which reinforces the validity of our conclusion. Also, since 2016, new drugs have been reimbursed and were not considered in this study. However, there are still cases of benign MS that do not require any treatment^12^. Therefore, our conclusions are unlikely to change. Finally, missing data could have affected our secondary analyses, so we took them into account using multiple imputation.

This work provides a clearer picture of the limitations of ADs and the potential impact their use may have on MS studies. Indeed, depending on their objective, their use may lead to risk of error in results. Therefore, ADs, although beneficial to investigate MS, should be used with caution. Moreover, MS registries collect rich and high-quality data that is impossible to obtain from ADs. Finally, by highlighting the value of MS registries that allow for a more exhaustive and rapid identification of cases, our findings support their development and the justification of the resources allocated.

### Supplementary Information

Supplementary Information.

## Data Availability

All data generated or analyzed during this study are included in this article and its supplementary material files. Further enquiries can be directed to the corresponding author.
