# Supplementary material for: Performance of administrative databases for identifying individuals with multiple sclerosis
Source: Sci Rep. 2023 Oct 25;13:18310. doi: 10.1038/s41598-023-45384-w (PMC10600163; doi:10.1038/s41598-023-45384-w)
Supplement: Supplementary file 1 — Supplementary Information. [file 41598_2023_45384_MOESM1_ESM.pdf]

Supplementary information

Performance of administrative databases for identifying individuals with multiple sclerosis

Pauline DUCATEL[1,2], Marc DEBOUVERIE[1,3], Marc SOUDANT[2], Francis GUILLEMIN[2,3], Guillaume MATHEY[1,3], Jonathan EPSTEIN[2,3]

[1]Departement of Neurology, Nancy University Hospital, Nancy, France
[2]CIC-EC 1433, CHRU, Inserm, Université de Lorraine, Vandoeuvre-Lès-Nancy, France
[3] Université de Lorraine, EA 4360 Apemac, Vandoeuvre-Lès-Nancy, France

Corresponding author: Pauline DUCATEL, p.ducatel@chru-nancy.fr, ORCID: 0000-0001-6129-3204

Supplementary information SI1: Table, data available for TP, FP, FN

Variables	TP	FP	FN
Age when identified by AD	X	X	
Age at onset	X		X
Sex	X	X	X
Department	X	X	X
First health care facility	X	X	X
Year of identification by the AD	X	X	
First EDSS	X		X
First brain MRI	X		X
CSF analysis performed	X		X
Number of relapses over the first 2 years	X		X
Initial treatment	X		X
Associated comorbidities	X		X

TP = true positive, FP = false positive, FN = false negative

Supplementary information SI2: Text, description of the hierarchical logistic regression models

Model 1: Factors associated with failure to identify multiple sclerosis by the administrative database. Results reported in Table 1.

- Dataset: true positives + false negatives
- Dependent variable: status
- Independent variables:
  - Fixed: age at onset, sex, first healthcare facility, associated comorbidities, first EDSS, Number of relapses over the first 2 years, first brain MRI, CSF analysis performed, initial treatment
  - Random: department

Model 2: Factors associated with misidentification as multiple sclerosis by the administrative database. Results reported in Table 3

- Dataset: true positives + false positives
- Dependent variable: status
- Independent variables:
  - Fixed: age at onset, sex
  - Random: department, year of identification by the AD

Model 3: Factors associated with early identification of individuals with multiple sclerosis by the administrative database. Results reported in Table 4.

- Dataset: true positives
- Dependent variable: first identification
- Independent variables:
  - Fixed: age at onset, sex, first healthcare facility, associated comorbidities, first EDSS, Number of relapses over the first 2 years, form of MS, first brain MRI, CSF analysis performed, initial treatment
  - Random: department, year of identification by the AD

Supplementary information SI3: Figure, Missing data pattern for true positives and false positives

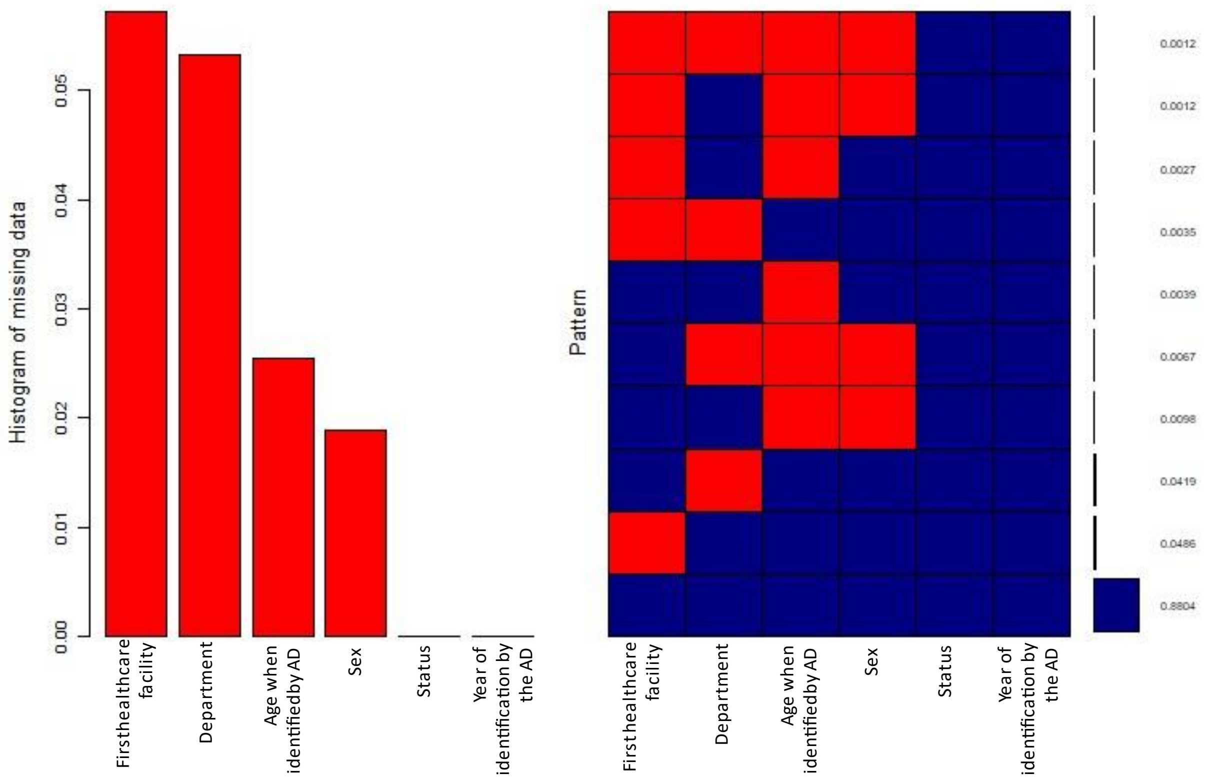

Supplementary information SI4: Figure, Missing data pattern for true positives and false negatives

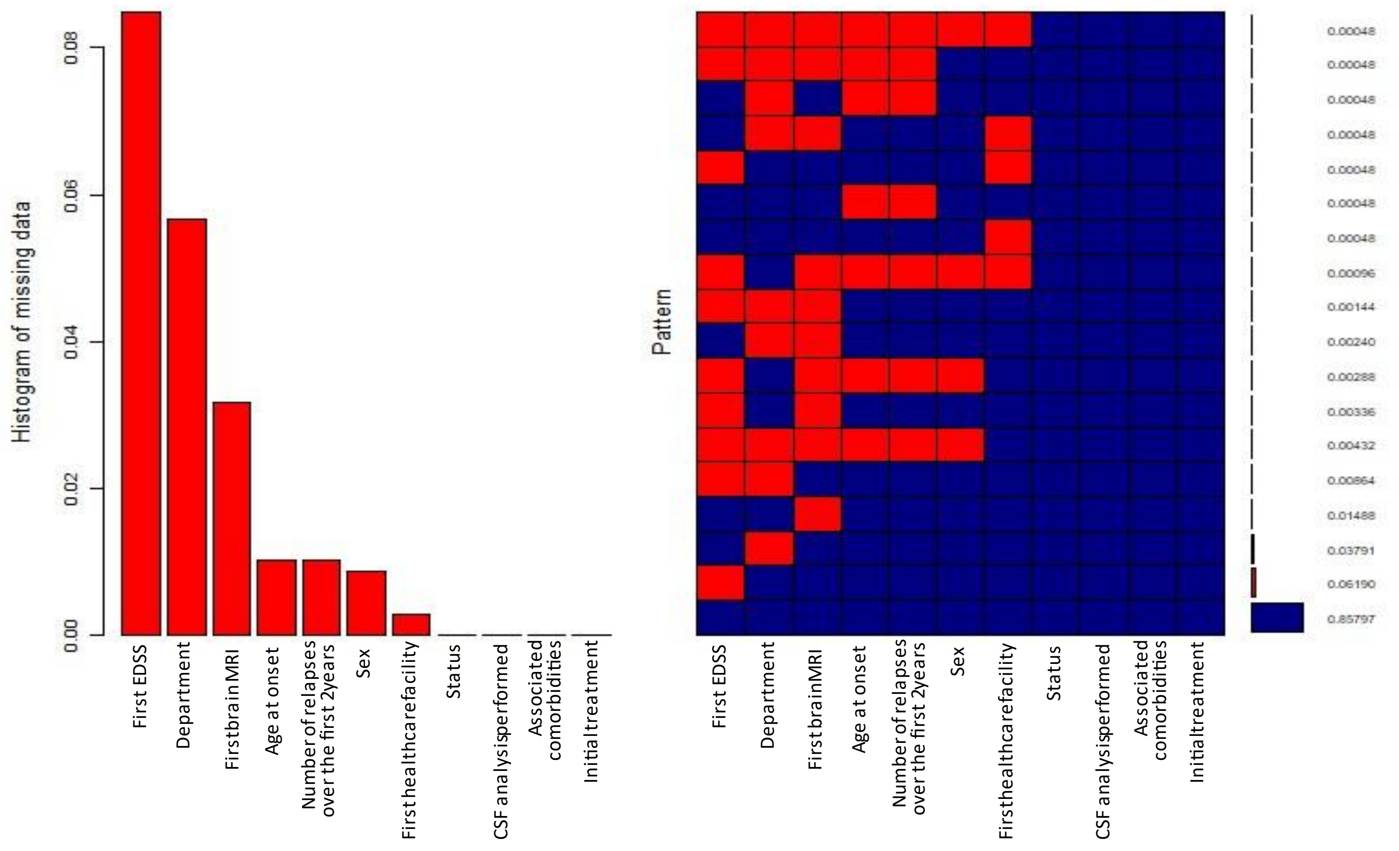

Supplementary information SI5: Figure, Missing data pattern for true positives

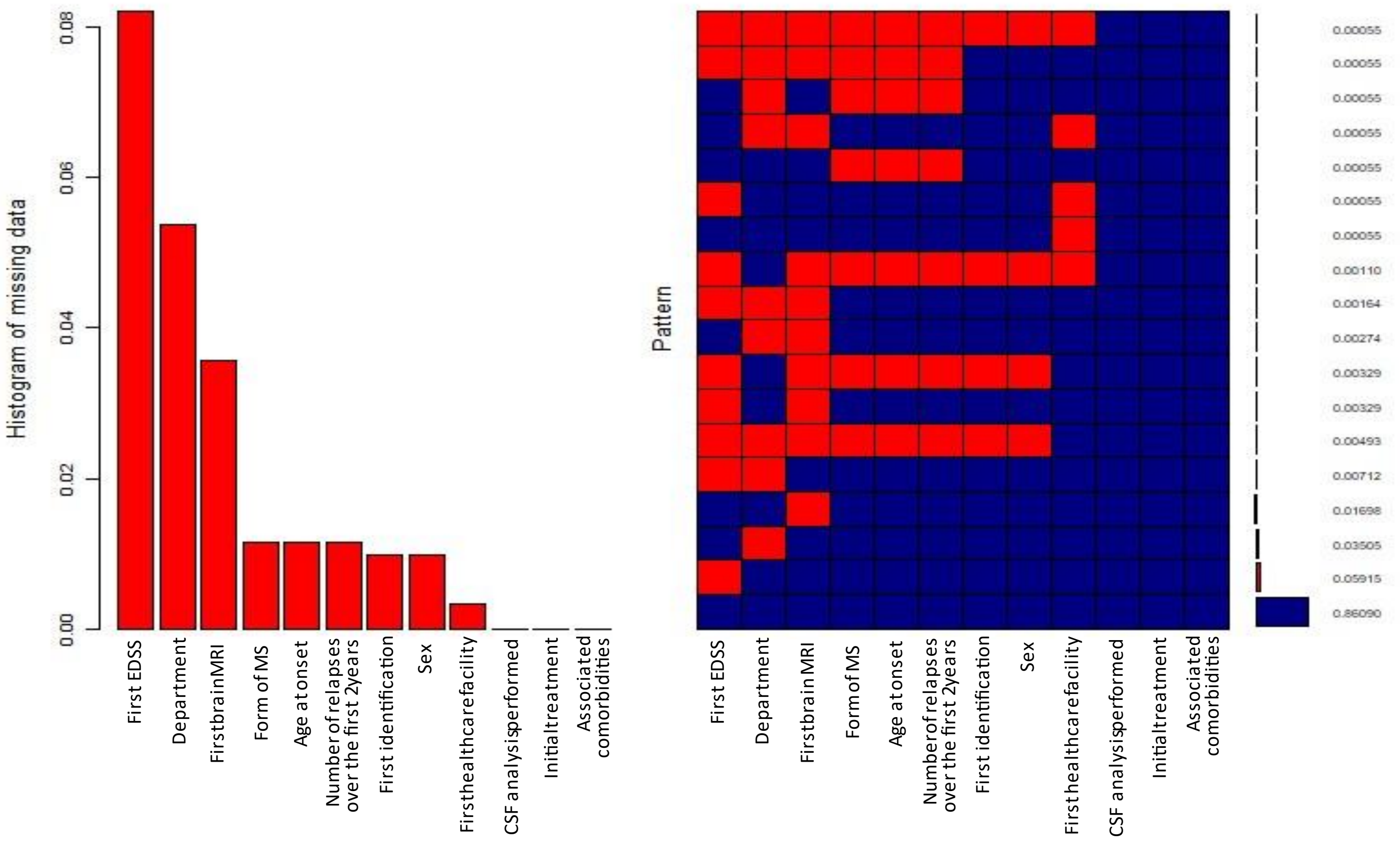

Supplementary information SI6: Contingency table, identification of individuals with MS by the administrative database and ReLSEP reference

	**ReLSEP +**	**ReLSEP -**	Total
**AD +**	True positive **1 826**	False positive **725**	2 551
**AD -**	False negative **258**	True negative **2 245 196**	2 245 454
Total	2 084	2 245 921	2 248 005

ReLSEP = “Registre Lorrain de la Sclérose en plaques”, AD = administrative database

Supplementary information SI7: Table, description of true positives and false negatives

Status		TP	FN	p
		N=1826	N=258	
Age at onset, years	Mean (SD)	33.4 (11.4)	33.5 (12.0)	0.880
Sex	Male	538 (29.8)	66 (25.6)	0.191
	Female	1270 (70.2)	192 (74.4)	
	(Missing)	18	0	
Department	54	511 (29.6)	83 (34.9)	**<0.001**
	55	115 (6.7)	19 (8.0)	
	57	728 (42.1)	69 (29.0)	
	88	270 (15.6)	27 (11.3)	
	Other	104 (6.0)	40 (16.8)	
	(Missing)	98	20	
First health care facility	Hospital	1409 (77.4)	215 (83.3)	**0.038**
	Private practice	411 (22.6)	43 (16.7)	
	(Missing)	6	0	
First EDSS	Mean (SD)	2.7 (2.0)	1.8 (1.6)	**<0.001**
First brain MRI	Normal	83 (4.7)	28 (10.9)	**<0.001**
	Abnormal	1678 (95.3)	229 (89.1)	
	(Missing)	65	1	
CSF analysis performed	Yes	1312 (71.9)	208 (80.6)	**0.004**
	No	514 (28.1)	50 (19.4)	
Number of relapses over the first 2 years	Mean (SD)	1.2 (0.9)	1.1 (0.6)	0.146
Initial treatment	None	315 (17.3)	88 (34.1)	**<0.001**
	Moderate	1211 (66.3)	139 (53.9)	
	Highly	300 (16.4)	31 (12.0)	
Associated comorbidities	No	611 (33.5)	104 (40.3)	**0.036**
	Yes	1215 (66.5)	154 (59.7)	

MS = multiple sclerosis, EDSS = Expended Disability Status Scale, CSF = cerebrospinal fluid, 54 = Meurthe et Moselle, 55 = Meuse, 57 = Moselle, 88 = Vosges, TP = true positive, FN = false negative

Supplementary information SI8: Table, description of true positives and false positives

Status		TP	FP	p
		N=1826	N=725	
Age when identified by AD, years	Mean (SD)	43.4 (15.0)	50.8 (18.6)	<0.001
Sex	Male	538 (29.8)	219 (31.5)	0.420
	Female	1270 (70.2)	476 (68.5)	
	(Missing)	18	30	
Department	54	511 (29.6)	135 (19.7)	<0.001
	55	115 (6.7)	54 (7.9)	
	57	728 (42.1)	342 (49.8)	
	88	270 (15.6)	83 (12.1)	
	Other	104 (6.0)	73 (10.6)	
	(Missing)	98	38	
First health care facility	Hospital	1409 (77.4)	469 (80.2)	0.179
	Private practice	411 (22.6)	116 (19.8)	
	(Missing)	6	140	
Year of identification by the AD	2009	19 (1.0)	0 (0.0)	<0.001
	2010	4 (0.2)	0 (0.0)	
	2011	389 (21.3)	143 (19.7)	
	2012	340 (18.6)	138 (19.0)	
	2013	259 (14.2)	108 (14.9)	
	2014	221 (12.1)	96 (13.2)	
	2015	293 (16.0)	118 (16.3)	
	2016	254 (13.9)	121 (16.7)	
	2017	47 (2.6)	1 (0.1)	

AD = administrative database, TP= true positive, FP = false positives, 54 = Meurthe et Moselle, 55 = Meuse, 57 = Moselle, 88 = Vosges

Supplementary information SI9: Tables, sensitivity analysis (primary analysis without the Moselle department)

	**ReLSEP +**	**ReLSEP -**	Total
**AD +**	True positive **1 000**	False positive **345**	1 345
**AD -**	False negative **169**	True negative **1 203 701**	1 203 870
Total	1 169	1 204 046	1 205 215

ReLSEP = “Registre Lorrain de la Sclérose en plaques”, AD = administrative database

**Sensitivity (95% CI)**	**PPV (95% CI)**	**F1-score (95% CI)**	**MCC (95% CI)**
0.86 [0.83-0.88]	0.74 [0.72-0.77]	0.80 [0.78-0.81]	0.79 [0.78-0.80]

Supplementary information SI10: Figure, Identification of false positives and true positives according to the 3 administrative database criteria

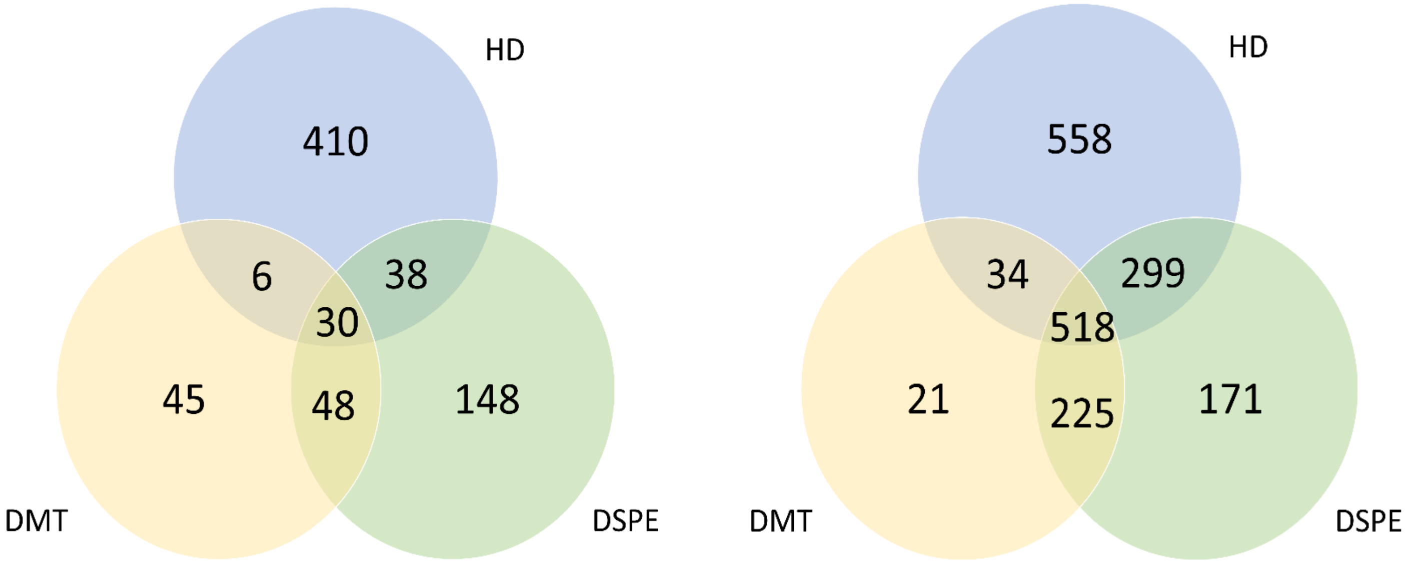

False positives

True positives

DMT = disease-modifying treatment, DSPE = disease-specific payment exemption, HD = hospital discharge

Supplementary information SI11: Figure, difference in identification time between ReLSEP and the administrative database in mutually recognized individuals

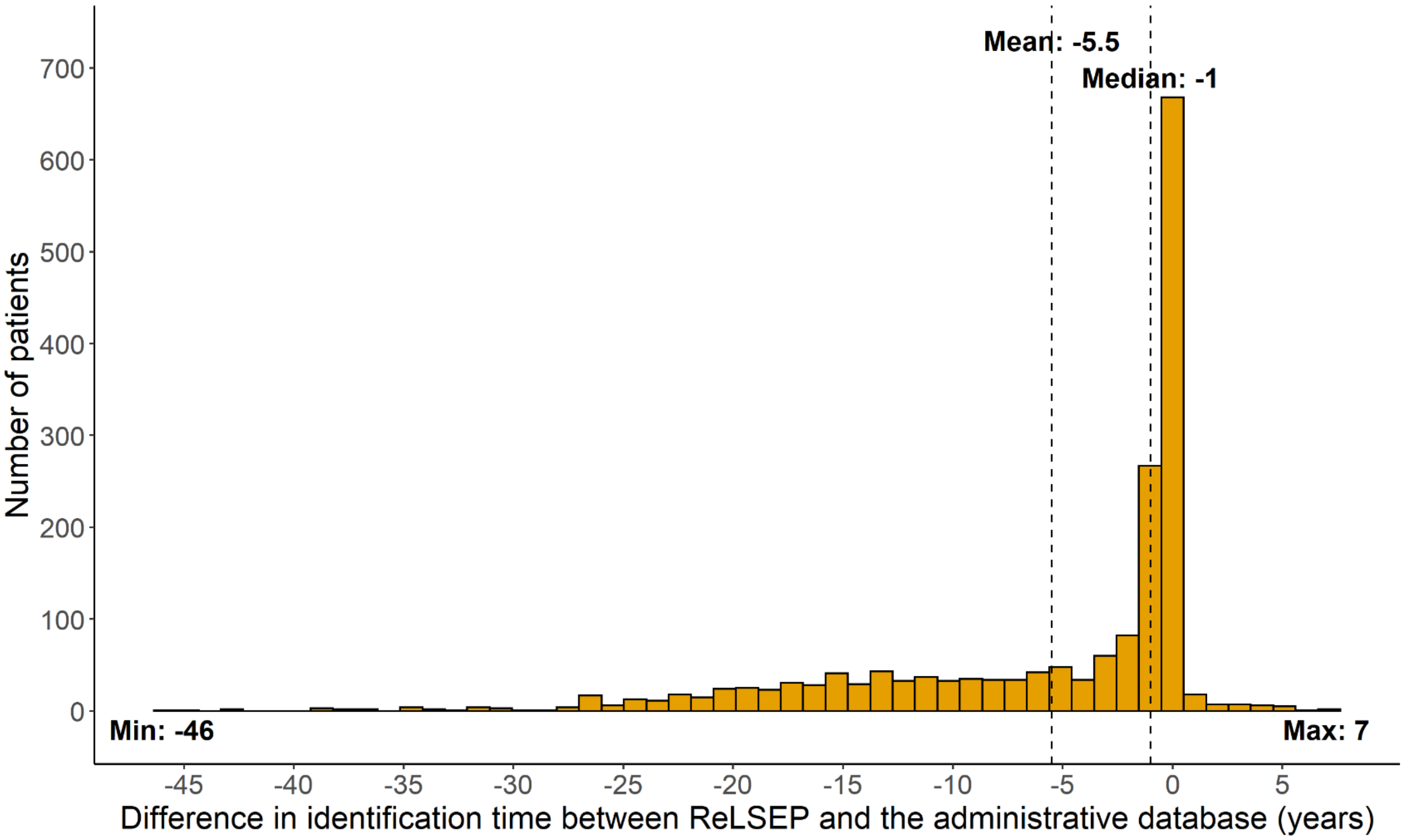

Supplementary information SI12: Table, Complete-case analysis, factors associated with misidentification as multiple sclerosis by the administrative database

Status		TP	FP	OR (multilevel imputed) (95% CI, p value)
		N=1718	N=528	
Age at onset, years	Mean (SD)	42.8 (14.7)	50. (18.3)	**1.03 (1.03-1.04, p<0.001)**
Sex	Male	507 (29.5)	172 (32.6)	-
	Female	1211 (70.5)	356 (67.4)	0.95 (0.77-1.18, p=0.827)

TP = true positive, FP = false positive, OR = odds ratio, 95% CI = 95% confidence interval

Supplementary information SI13: Table, Complete-case analysis, factors associated with failure to identify multiple sclerosis by the administrative database

Status		TP	FN	OR (multilevel imputed) (95% CI, p value)
		N=1572	N=216	
Age at onset, years	Mean (SD)	33.3 (11.3)	32.7 (11.9)	1.00 (0.99-1.02, p=0.952)
Sex	Male	466 (29.8)	55 (25.5)	-
	Female	1106 (70.4)	161 (74.5)	1.04 (0.74-1.48, p=0.812)
First healthcare facility	Hospital	1212 (77.1)	181 (83.8)	-
	Private practice	360 (22.9)	35 (16.2)	0.68 (0.45-1.01, p=0.057)
Associated comorbidities	No	512 (32.6)	87 (40.3)	-
	Yes	1060 (67.4)	129 (59.7)	0.83 (0.61-1.14, p=0.257)
First EDSS	Mean (SD)	2.6 (1.9)	1.7 (1.6)	**0.67 (0.61-0.75, p<0.001)**
Number of relapses over the first 2 years	Mean (SD)	1.2 (0.8)	1.1 (0.5)	**0.61 (0.46-0.79, p<0.001)**
First brain MRI	Normal	66 (4.2)	19 (8.8)	-
	Abnormal	1506 (95.8)	297 (91.2)	**0.47 (0.26-0.84, p=0.011)**
CSF analysis performed	Yes	1176 (74.8)	176 (81.5)	-
	No	396 (25.2)	40 (18.5)	0.82 (0.55-1.21, p=0.318)
Initial treatment	None	202 (12.8)	62 (38.7)	-
	Moderate	1102 (70.1)	129 (59.7)	**0.33 (0.22-0.48, p<0.001)**
	Highly	268 (17.0)	25 (11.6)	**0.42 (0.24-0.72, p=0.002)**

EDSS = Expended Disability Status Scale, CSF = cerebrospinal fluid, TP = false positive, FN = true negative, OR = odds ratio, 95% CI = 95% confidence interval

Supplementary information SI14: Table, Complete-case analysis, factors associated with early identification of individuals with multiple sclerosis by the administrative database

**First identification**		**ReLSEP**	**AD**	**OR (multilevel imputed) (95% CI, p value)**
		N=925	N=647	
Age at onset, years	Mean (SD)	33.5 (11.4)	32.9 (11.2)	1.00 (0.99-1.01, p=0.695)
Sex	Male	254 (27.5)	212 (32.8)	-
	Female	671 (72.5)	435 (67.2)	**0.70 (0.54-0.90, p=0.006)**
First healthcare facility	Hospital	673 (72.8)	539 (83.3)	-
	Private practice	252 (27.2)	108 (16.7)	**0.57 (0.43-0.75, p<0.001)**
Associated comorbidities	No	305 (33.0)	207 (32.0)	-
	Yes	620 (67.0)	440 (68.0)	0.98 (0.77-1.26, p=0.888)
First EDSS	Mean (SD)	2.8 (2.0)	2.4 (1.8)	1.05 (0.96-1.14, p=0.301)
Number of relapses over the first 2 years	Mean (SD)	1.2 (0.8)	1.3 (0.8)	1.12 (0.95-1.33, p=0.174)
Form of MS	RR	575 (62.2)	534 (82.5)	-
	PP	135 (14.6)	92 (14.2)	**0.55 (0.33-0.91, p=0.020)**
	SP	215 (23.2)	21 (3.2)	**0.08 (0.04-0.14, p<0.001)**
First brain MRI	Normal	55 (5.9)	11 (1.7)	-
	Abnormal	870 (94.1)	636 (98.3)	**4.38 (2.20-8.71, p<0.001)**
CSF analysis performed	Yes	627 (67.8)	549 (84.9)	-
	No	298 (32.2)	98 (15.1)	**0.45 (0.34-0.61, p<0.001)**
Initial treatment	None	145 (15.7)	57 (8.8)	-
	Moderate	634 (68.5)	468 (72.3)	**1.65 (1.11-2.44, p=0.012)**
	Highly	146 (15.8)	122 (18.9)	**3.16 (1.98-5.05, p<0.001)**

MS = multiple sclerosis, EDSS = Expended Disability Status Scale, CSF = cerebrospinal fluid, AD= administrative database, ReLSEP = *Registre Lorrain de la Sclérose en plaques*, RR = relapse remitting, PP = primary progressive, SP = secondary progressive, OR = odds ratio, 95% CI = 95% confidence interval
